# Detainer Requests Issued by ICE and Fair/Poor Self-Rated Health among Latines in the U.S., 2017–2020

**DOI:** 10.1007/s11524-024-00908-1

**Published:** 2024-09-09

**Authors:** Alexandra Eastus, Amy H. Auchincloss, M. Pia Chaparro, Sofia Argibay, Caroline Kravitz, Brent A. Langellier

**Affiliations:** 1 Department of Health Management and Policy, Dornsife School of Public Health, Drexel University, Nesbitt Hall, 3215 Market St., Philadelphia, PA 19104 USA; 2 Department of Epidemiology and Biostatistics, Dornsife School of Public Health, Drexel University, Nesbitt Hall, 3215 Market St., Philadelphia, PA 19104 USA; 3 Department of Health Systems and Population Health, School of Public Health, University of Washington, 306C Raitt Hall, Box 353410, Seattle, WA 98195 USA

**Keywords:** Immigration enforcement, Self-rated health, Health disparities, Policy

## Abstract

U.S. Immigration and Customs Enforcement (ICE) issues detainer requests to local law enforcement agencies to hold detainees suspected of being undocumented immigrants until they can be transferred into ICE custody. We examined the association between area-level detainer requests and self-rated health among Latine adults*.* We linked health data from Latine adults included in the 2017–2020 Behavioral Risk Factor Surveillance System (*n* = 69,386) to detainer requests per 1,000 non-citizens in core-based statistical areas, (*n* = 152 across 49 states). We fit logistic regression models of self-rated fair/poor health on detainer requests, adjusted for individual- and area-level confounders. In adjusted analyses, we found that Latine adults living in areas with the highest quartile of requests had 24% higher odds of fair/poor health (OR 1.24, 95% CI = 1.05,1.47) relative to those in the lowest quartile*.* Local law enforcement agencies should limit cooperation with federal immigrant agencies to protect the health of Latine communities.

**Supplementary Information:**

The online version contains supplementary material available at 10.1007/s11524-024-00908-1.

## Introduction

Local enforcement of federal immigration policies can contribute to a pervasive culture of fear within immigrant communities and impact health-related outcomes. For example, U.S. Immigration and Customs Enforcement (ICE) issues I-247 detainer requests to local law enforcement agencies to temporarily detain individuals suspected of being undocumented until ICE agents can assume custody [1]. ICE cannot mandate that local agencies comply with these requests; [1] in fact, some localities implement “sanctuary” policies to limit the extent by which local officers can cooperate with ICE. In areas that do comply with detainer requests, this type of federal-local coordination increases deportation risk because the number of state and local law enforcement officers vastly exceeds the number of ICE agents, and relatively low-level interactions with law enforcement can lead to deportation risk [2]. Although detainer requests are conducted in jail settings and do not always result in arrest or deportation, they directly impact how racialized communities interact with law enforcement agencies. This can lead to a culture of fear and mental distress among undocumented immigrants, authorized immigrants, and U.S. citizens who have close social ties to undocumented individuals [2–4]. Prior research suggests that immigration enforcement can decrease utilization of health and social services, creating a “chilling effect” on enrollment in public programs among eligible immigrants and citizens with undocumented immigrants in their families or social networks [5]. While any racial/ethnic group can experience chilling effects, recent anti-immigrant rhetoric and racial profiling under the guise of immigration enforcement has targeted Latines [6, 7].

Over two-thirds of undocumented immigrants in the U.S. are from Mexico or Central America, making the Latine community an important demographic population for immigration policy and enforcement [8]. Furthermore, prior research has established that immigrant policies and enforcement impact Latine populations, including undocumented immigrants as well as the broader populations of authorized immigrants and Latine citizens [2, 9, 10]. A broad set of policies criminalizing immigrants were associated with an increase in self-reported mental health distress and fair or poor (vs. good to excellent) health among Latines [3]. Further, county-level food assistance participation among Latine households was reported to be higher in counties and states with policies that integrate rather than criminalize immigrants [9]. Latines in areas with greater levels of detainer requests were less likely to report having a regular healthcare provider [5]. Collectively, fear and stress related to immigration enforcement, as well as foregone care and under-enrollment in public assistance programs, can impact the physical and mental health of the Latine population [5, 10].

No prior research has directly assessed the relationship between area-level immigration enforcement and self-rated health among Latines. In the context of immigration enforcement, detainer requests represent functions of policing and policy coordination with ICE. Although local compliance with detainer requests is unknown, detainer requests are harmful because they directly influence the threat of arrest and deportation. We addressed this gap using administrative data on the issuance of detainer requests and self-rated health among Latines using nationally representative data. We hypothesized that Latines in areas with greater exposure to immigration enforcement would have higher odds of reporting fair/poor health versus good/excellent health as compared to those in areas with less enforcement.

## Methods

We used individual-level data from the 2017–2020 Selected Metropolitan/Micropolitan Area Risk Trends of the Behavioral Risk Factor Surveillance System (SMART BRFSS), which included a geographic identifier for each respondent’s core-based statistical area (CBSA) [11]. CBSAs are economically and socially integrated counties centered around an urban core and are commonly used for federal resource allocation. We excluded respondents self-reporting as non-Hispanic/Latine, those who lived in Puerto Rico, and those missing data for the exposure, the outcome, or individual-level covariates. In total, our analytic sample included 69,386 adult respondents living in 152 CBSAs (142 metropolitan) across 49 states in 2017–2020 (see Supplementary Appendix).

### Measures

#### Outcome

The outcome was a binary variable indicating fair or poor self-rated health vs. excellent, very good, or good health. Self-rated health has shown to be reliable across cultures, age groups, and community settings, [12] and to predict physical and mental health outcomes [12].

#### Exposure

The main exposure was the number of ICE detainer requests issued per 1000 non-citizens in the CBSA. These data are from the Syracuse University Transactional Records Access Clearinghouse (TRAC) [1]. We conducted a Box-Tidwell test and plotted the log odds of detainer requests against the original predictor and observed non-linearity. Due to this non-linearity, we classified the exposure into quartiles by year. We chose quartiles, rather than dichotomizing at the median, to preserve variation and because model fit was better with quartiles vs. the binary categorization.

#### Confounders

We adjusted for potential confounders that could plausibly impact both individuals’ selection into CBSAs (primarily residential mobility) and self-rated health, including age, sex, marital status, household income, education, employment status, health insurance coverage, having a regular health provider, and CBSA-level poverty. Conceptually, we believe that area-level poverty impacts the number of detainer requests because law enforcement has historically policed marginalized, often low-income, communities at a disproportionate rate. Other CBSA-level variables (proportion non-citizen, proportion of population undocumented, and distance to U.S.-Mexico border) were included in the model but removed due to collinearity with other predictors.

### Statistical Analysis

In all analyses, we used raking sampling weights provided by SMART BRFSS to account for unequal selection and nonresponse. We calculated descriptive statistics across levels of detainer request quartiles. Further, we fit a multivariable logistic regression model of fair/poor self-rated health on quartiles of detainer requests, adjusted for confounders. We included state and year-fixed effects to account for unmeasured confounding.

## Results

Between 2017 and 2020, there were approximately 465,213 detainer requests per 1000 non-citizens issued in the 152 CBSAs included in the study; the median number of detainer requests was 33 (25th–75th percentile: 7–89). Unadjusted for other factors, there were no strong patterns in sociodemographic variables by detainer request quartiles (see Supplementary Appendix). Across the entire sample, half of the participants reported being male and between the ages of 18 and 39 years old. Approximately 33% did not graduate high school, 60% were employed for wages, and 33% had income less than $25,000.

In adjusted analyses, Latine adults living in metropolitan areas in the highest quartile of detainer requests had 24% higher odds of fair/poor self-rated health relative to those in the lowest quartile (OR 1.24, 95% CI = 1.05, 1.47, Table 1).

**Table 1:** Odds ratios of fair/poor health according to quartiles of detainer requests

ICE Detainer Requests Issued per 1,000 Non-Citizens****	Unadjusted OR [95% CI]	Fully Adjusted OR^1^ [95% CI]
Lowest Quartile 1	ref	ref
Low Quartile 2	1.16 [1.05, 1.28]	1.20 [1.05, 1.38]
High Quartile 3	1.12 [1.03, 1.22]	1.19 [0.99, 1.42]
Highest Quartile 4	1.28 [1.16, 1.43]	1.24 [1.05, 1.47]

**** Quartiles for ICE detainer requests calculated per year. [2017] Quartile 1—Lowest < 6.50; Quartile 3—High > 40.19 <  = 92.35. [2018] Quartile 1—Lowest < 6.14; Quartile 3—High > 40.44 <  = 103.29. [2019] Quartile 1—Lowest < 11.93; Quartile 3—High > 32.36 <  = 110.74. [2020] Quartile 1—Lowest < 6.73; Quartile 3—High > 29.15 <  = 48.97

^1^Adjusted for age, sex, marital status, individual income, education, employment status, health insurance coverage, medial home, and proportion of area reporting poverty

## Discussion

Our findings show that area-level detainer requests are negatively associated with self-reported health among Latine adults, which is directionally consistent with prior research focused on the impact of immigrant policies on health [9, 10, 13]. Specifically, our findings add to existing evidence that immigration enforcement policies and practices are harmful to the health of the broad Latine population, rather than the more narrow group of undocumented immigrants that are typically the “direct” targets of immigration enforcement and criminalizing policies [3, 10]. Both our descriptive findings—illustrating that the number of detainer requests does not meaningfully vary by socioeconomic status (SES)—and our multivariable results—adjusted for household- and area-level SES—suggest that a higher SES does not protect against harmful policing and immigration practices. Detainer requests may be especially harmful because ICE has historically issued them to a high number of individuals without serious criminal convictions, who are typically not considered a threat to local or national security [14]. This form of racialized over-policing may instill fear within immigrant communities during interactions with law enforcement because of a perceived threat of deportation, which can negatively impact health.

### Strengths and Limitations

A strength of our study is that we directly examined detainer requests as functions of policing and policy coordination between local law enforcement and ICE, whereas many prior studies proxied immigration enforcement exposure by assessing the presence or absence of state or federal policies that may impact local enforcement levels (e.g., 287(g) legislation or local agreements, sanctuary policies) [3]. A second strength is that we examined self-rated health, which has been shown to accurately predict mortality and morbidity [12]. Most prior studies examined outcomes that drive population health (e.g., healthcare access, public program participation) but are not direct health indicators [5, 9].

A limitation is that information about individuals’ immigration status is not collected by BRFSS. Thus, we cannot differentiate between direct impacts of immigration enforcement on undocumented immigrants and spillover effects on authorized immigrants or U.S.-born Latines. Another limitation is that we do not know the proportion of detainer requests that end in custody transfer to ICE, arrest, or deportation; this information is withheld by ICE. While arrest and removal likely have additional health implications, issuance of detainer requests is a widespread mechanism that contributes to a culture of fear and impacts the degree to which individuals and communities are racialized and policed. Further, due to limits on the disclosure of geographic identifiers in SMART BRFSS, our findings are not generalizable to rural CBSAs. Finally, our models may not account for all potential individual- or area-level confounders.

## Conclusion

Our findings suggest that the self-rated health of Latines may be improved by reducing exposure to high levels of detainer requests. Local law enforcement should limit policing and policy coordination with federal immigration agencies to protect the health of Latine communities. This could address health inequities that affect historically marginalized immigrant communities.

## Supplementary Information

Below is the link to the electronic supplementary material.

Supplementary file1 (DOCX 38 KB)

## Data Availability

Data are available on ICPSR. Project citation: Eastus, Alexandra. Detainer Requests Issued by ICE and Fair/Poor Self-Rated Health Among Latines in the United States, 2017–2020. Ann Arbor, MI: Inter-university Consortium for Political and Social Research [distributor], 2024-03-04. 10.3886/E198805V1.
