# Supplementary material for: Detainer Requests Issued by ICE and Fair/Poor Self-Rated Health among Latines in the U.S., 2017–2020
Source: J Urban Health. 2024 Sep 9;102(1):3–7. doi: 10.1007/s11524-024-00908-1 (PMC11865394; doi:10.1007/s11524-024-00908-1)
Supplement: Supplementary file 1 — Supplementary file1 (DOCX 38 KB) [file 11524_2024_908_MOESM1_ESM.docx]

**Supplementary Table 1.** Descriptive statistics of weighted SMART BRFSS sample 2017 - 2020* by quartile of detainer requests*

|  | Lowest Quartile 1  n = 17,118 | Low Quartile 2 n = 17,256 | Higher Quartile 3 n = 18,170 | Highest Quartile 4 n = 16,842 |
| --- | --- | --- | --- | --- |
| **Fair/Poor General Health Status (%)** | 25.84 | 23.10 | 23.78 | 21.34 |
| **Sex (%)** |  |  |  |  |
| Male | 49.28 | 49.14 | 49.37 | 50.18 |
| Female | 50.72 | 50.86 | 50.63 | 49.82 |
| **Age (%)** |  |  |  |  |
| Age 18 to 29 | 25.34 | 25.59 | 27.21 | 29.73 |
| Age 30 to 39 | 22.89 | 22.24 | 24.38 | 25.16 |
| Age 40 to 49 | 18.53 | 19.01 | 19.15 | 18.76 |
| Age 50 to 59 | 15.64 | 15.50 | 14.40 | 13.01 |
| Age 60 to 69 | 10.09 | 9.68 | 8.94 | 8.44 |
| Age 70 to 79 | 7.52 | 7.61 | 5.91 | 4.89 |
| **Marital Status (%)** |  |  |  |  |
| Currently married | 42.23 | 44.94 | 42.06 | 45.85 |
| Not currently married | 17.44 | 18.62 | 18.19 | 16.25 |
| Never Married | 39.33 | 36.44 | 39.75 | 37.90 |
| **Educational Attainment (%)** |  |  |  |  |
| Did not graduate High School | 36.60 | 29.18 | 34.77 | 33.04 |
| Graduated High School | 25.86 | 28.35 | 26.39 | 27.22 |
| Attended College or Technical School | 24.28 | 24.83 | 22.48 | 24.04 |
| Graduated from College or Technical School | 13.26 | 17.64 | 16.35 | 15.69 |
| **Employment Status (%)** |  |  |  |  |
| Employed for wages | 59.07 | 61.27 | 61.50 | 63.27 |
| Unemployed | 8.45 | 7.10 | 8.89 | 7.00 |
| Homemaker | 10.99 | 9.77 | 9.85 | 11.99 |
| Student | 5.60 | 6.03 | 5.61 | 5.46 |
| Unable to work | 9.07 | 9.44 | 7.28 | 6.81 |
| Retired | 6.81 | 6.40 | 6.87 | 5.47 |
| **Income (%)** |  |  |  |  |
| Less than $15,000 | 18.43 | 13.74 | 13.20 | 12.22 |
| $15,000 to less than $25,000 | 20.01 | 18.52 | 22.07 | 21.50 |
| $25,000 to less than $35,000 | 10.81 | 11.38 | 9.57 | 10.08 |
| $35,000 to less than $50,000 | 10.28 | 10.80 | 9.89 | 10.83 |
| $50,000 or more | 24.73 | 26.51 | 24.48 | 27.09 |
| Income Data Missing | 15.74 | 19.05 | 20.79 | 18.38 |
| **Does have health insurance coverage (%)** | 74.98 | 76.63 | 67.86 | 62.20 |
| **Does not have a personal doctor or provider (%)** | 35.38 | 34.37 | 40.10 | 45.92 |
| **Proportion of CBSA in Poverty** [Median (IQR)**] | 13.20 (12.10, 13.90) | 12.80 (12.10, 13.90) | 11.80 (11.10, 12.40) | 13.30 (11.20, 13.90) |
| *** ICE Detainer Requests Issued Per 1,000 Non-Citizens: Quartiles for ICE detainer requests calculated per year. [2017]** Quartile 1 - Lowest < 6.50; Quartile 2 - Low > 6.50 <= 40.19; Quartile 3 - High > 40.19 <= 92.35; Quartile 4 - Highest > 92.35. **[2018]** Quartile 1 - Lowest < 6.14; Quartile 2 - Low > 6.14 <= 40.44; Quartile 3 - High > 40.44 <= 103.29; Quartile 4 - Highest > 103.29. **[2019]** Quartile 1 - Lowest < 11.93; Quartile 2 - Low > 11.93 <= 32.36; Quartile 3 - High > 32.36 <= 110.74; Quartile 4 - Highest > 110.74. **[2020**] Quartile 1 - Lowest < 6.73; Quartile 2 - Low > 6.73 <= 29.15; Quartile 3 - High > 29.15 <= 48.97; Quartile 4 - Highest > 48.97**. ** Interquartile Range (IQR)** | | | | |

**Supplementary Table 2.** Participant core based statistical area (CBSA), by state and CBSA type. Metropolitan/Micropolitan Area Risk Trends of the Behavioral Risk Factor Surveillance System (SMART BRFSS) 2017-2020. All states except Wyoming were included in the sample.

| **CBSA Code** | **CBSA Name** | **State** | **CBSA Type** |
| --- | --- | --- | --- |
| 11260 | Anchorage | AK | Metropolitan Statistical Area |
| 13820 | Birmingham-Hoover | AL | Metropolitan Statistical Area |
| 33660 | Mobile | AL | Metropolitan Statistical Area |
| 33860 | Montgomery | AL | Metropolitan Statistical Area |
| 46220 | Tuscaloosa | AL | Metropolitan Statistical Area |
| 30780 | Little Rock-North Little Rock-Conway | AR | Metropolitan Statistical Area |
| 22220 | Fayetteville-Springdale-Rogers | AR-MO | Metropolitan Statistical Area |
| 38060 | Phoenix-Mesa-Scottsdale | AZ | Metropolitan Statistical Area |
| 31080 | Los Angeles-Long Beach-Anaheim | CA | Metropolitan Statistical Area |
| 41860 | Oakland-Hayward-Berkeley | CA | Metropolitan Division |
| 40140 | Riverside-San Bernardino-Ontario | CA | Metropolitan Statistical Area |
| 40900 | Sacramento--Roseville--Arden-Arcade | CA | Metropolitan Statistical Area |
| 41940 | San Jose-Sunnyvale-Santa Clara | CA | Metropolitan Statistical Area |
| 17820 | Colorado Springs | CO | Metropolitan Statistical Area |
| 19740 | Denver-Aurora-Lakewood | CO | Metropolitan Statistical Area |
| 25540 | Hartford-West Hartford-East Hartford | CT | Metropolitan Statistical Area |
| 37980 | Wilmington | DE-MD-NJ | Metropolitan Division |
| 18880 | Crestview-Fort Walton Beach-Destin | FL | Metropolitan Statistical Area |
| 19660 | Deltona-Daytona Beach-Ormond Beach | FL | Metropolitan Statistical Area |
| 23540 | Gainesville | FL | Metropolitan Statistical Area |
| 27260 | Jacksonville | FL | Metropolitan Statistical Area |
| 33100 | Miami-Fort Lauderdale-West Palm Beach | FL | Metropolitan Statistical Area |
| 35840 | North Port-Sarasota-Bradenton | FL | Metropolitan Statistical Area |
| 36740 | Orlando-Kissimmee-Sanford | FL | Metropolitan Statistical Area |
| 37460 | Panama City | FL | Metropolitan Statistical Area |
| 37860 | Pensacola-Ferry Pass-Brent | FL | Metropolitan Statistical Area |
| 38940 | Port St. Lucie | FL | Metropolitan Statistical Area |
| 45220 | Tallahassee | FL | Metropolitan Statistical Area |
| 45300 | Tampa-St. Petersburg-Clearwater | FL | Metropolitan Statistical Area |
| 12060 | Atlanta-Sandy Springs-Roswell | GA | Metropolitan Statistical Area |
| 12260 | Augusta-Richmond County | GA-SC | Metropolitan Statistical Area |
| 27980 | Kahului-Wailuku-Lahaina | HI | Metropolitan Statistical Area |
| 16300 | Cedar Rapids | IA | Metropolitan Statistical Area |
| 19780 | Des Moines-West Des Moines | IA | Metropolitan Statistical Area |
| 47940 | Waterloo-Cedar Falls | IA | Metropolitan Statistical Area |
| 19340 | Davenport-Moline-Rock Island | IA-IL | Metropolitan Statistical Area |
| 43580 | Sioux City | IA-NE-SD | Metropolitan Statistical Area |
| 14260 | Boise City | ID | Metropolitan Statistical Area |
| 26820 | Idaho Falls | ID | Metropolitan Statistical Area |
| 16980 | Chicago-Naperville-Elgin | IL-IN-WI | Metropolitan Statistical Area |
| 23060 | Fort Wayne | IN | Metropolitan Statistical Area |
| 26900 | Indianapolis-Carmel-Anderson | IN | Metropolitan Statistical Area |
| 21780 | Evansville | IN-KY | Metropolitan Statistical Area |
| 43780 | South Bend-Mishawaka | IN-MI | Metropolitan Statistical Area |
| 31740 | Manhattan | KS | Metropolitan Statistical Area |
| 41460 | Salina | KS | Micropolitan Statistical Area |
| 45820 | Topeka | KS | Metropolitan Statistical Area |
| 48620 | Wichita | KS | Metropolitan Statistical Area |
| 30460 | Lexington-Fayette | KY | Metropolitan Statistical Area |
| 31140 | Louisville/Jefferson County | KY-IN | Metropolitan Statistical Area |
| 12940 | Baton Rouge | LA | Metropolitan Statistical Area |
| 29180 | Lafayette | LA | Metropolitan Statistical Area |
| 35380 | New Orleans-Metairie | LA | Metropolitan Statistical Area |
| 14460 | Boston | MA | Metropolitan Division |
| 44140 | Springfield | MA | Metropolitan Statistical Area |
| 49340 | Worcester | MA-CT | Metropolitan Statistical Area |
| 12580 | Baltimore-Columbia-Towson | MD | Metropolitan Statistical Area |
| 47900 | Silver Spring-Frederick-Rockville | MD | Metropolitan Division |
| 41540 | Salisbury | MD-DE | Metropolitan Statistical Area |
| 19060 | Cumberland | MD-WV | Metropolitan Statistical Area |
| 25180 | Hagerstown-Martinsburg | MD-WV | Metropolitan Statistical Area |
| 38860 | Portland-South Portland | ME | Metropolitan Statistical Area |
| 24340 | Grand Rapids-Wyoming | MI | Metropolitan Statistical Area |
| 29620 | Lansing-East Lansing | MI | Metropolitan Statistical Area |
| 19820 | Warren-Troy-Farmington Hills | MI | Metropolitan Division |
| 40340 | Rochester | MN | Metropolitan Statistical Area |
| 41060 | St. Cloud | MN | Metropolitan Statistical Area |
| 20260 | Duluth | MN-WI | Metropolitan Statistical Area |
| 33460 | Minneapolis-St. Paul-Bloomington | MN-WI | Metropolitan Statistical Area |
| 41180 | St. Louis | MO-IL | Metropolitan Statistical Area |
| 28140 | Kansas City | MO-KS | Metropolitan Statistical Area |
| 25060 | Gulfport-Biloxi | MS | Metropolitan Statistical Area |
| 27140 | Jackson | MS | Metropolitan Statistical Area |
| 13740 | Billings | MT | Metropolitan Statistical Area |
| 39580 | Raleigh-Cary | NC | Metropolitan Statistical Area |
| 16740 | Charlotte-Concord-Gastonia | NC-SC | Metropolitan Statistical Area |
| 13900 | Bismarck | ND | Metropolitan Statistical Area |
| 33500 | Minot | ND | Micropolitan Statistical Area |
| 22020 | Fargo | ND-MN | Metropolitan Statistical Area |
| 24220 | Grand Forks | ND-MN | Metropolitan Statistical Area |
| 24260 | Grand Island | NE | Metropolitan Statistical Area |
| 30700 | Lincoln | NE | Metropolitan Statistical Area |
| 35740 | Norfolk | NE | Micropolitan Statistical Area |
| 35820 | North Platte | NE | Micropolitan Statistical Area |
| 42420 | Scottsbluff | NE | Micropolitan Statistical Area |
| 36540 | Omaha-Council Bluffs | NE-IA | Metropolitan Statistical Area |
| 17200 | Claremont-Lebanon | NH-VT | Micropolitan Statistical Area |
| 30100 | Lebanon | NH-VT | Micropolitan Statistical Area |
| 35620 | Newark | NJ-PA | Metropolitan Division |
| 10740 | Albuquerque | NM | Metropolitan Statistical Area |
| 39900 | Reno | NV | Metropolitan Statistical Area |
| 10580 | Albany-Schenectady-Troy | NY | Metropolitan Statistical Area |
| 13780 | Binghamton | NY | Metropolitan Statistical Area |
| 15380 | Buffalo-Cheektowaga-Niagara Falls | NY | Metropolitan Statistical Area |
| 24020 | Glens Falls | NY | Metropolitan Statistical Area |
| 40380 | Rochester | NY | Metropolitan Statistical Area |
| 45060 | Syracuse | NY | Metropolitan Statistical Area |
| 46540 | Utica-Rome | NY | Metropolitan Statistical Area |
| 10420 | Akron | OH | Metropolitan Statistical Area |
| 17460 | Cleveland-Elyria | OH | Metropolitan Statistical Area |
| 18140 | Columbus | OH | Metropolitan Statistical Area |
| 19380 | Dayton | OH | Metropolitan Statistical Area |
| 19430 | Dayton-Kettering | OH | Metropolitan Statistical Area |
| 45780 | Toledo | OH | Metropolitan Statistical Area |
| 17140 | Cincinnati | OH-KY-IN | Metropolitan Statistical Area |
| 49660 | Youngstown-Warren-Boardman | OH-PA | Metropolitan Statistical Area |
| 36420 | Oklahoma City | OK | Metropolitan Statistical Area |
| 46140 | Tulsa | OK | Metropolitan Statistical Area |
| 41420 | Salem | OR | Metropolitan Statistical Area |
| 38900 | Portland-Vancouver-Hillsboro | OR-WA | Metropolitan Statistical Area |
| 38300 | Pittsburgh | PA | Metropolitan Statistical Area |
| 10900 | Allentown-Bethlehem-Easton | PA-NJ | Metropolitan Statistical Area |
| 39300 | Providence-Warwick | RI-MA | Metropolitan Statistical Area |
| 16700 | Charleston-North Charleston | SC | Metropolitan Statistical Area |
| 17900 | Columbia | SC | Metropolitan Statistical Area |
| 22500 | Florence | SC | Metropolitan Statistical Area |
| 24860 | Greenville-Anderson-Mauldin | SC | Metropolitan Statistical Area |
| 25940 | Hilton Head Island-Bluffton-Beaufort | SC | Metropolitan Statistical Area |
| 43900 | Spartanburg | SC | Metropolitan Statistical Area |
| 34820 | Myrtle Beach-Conway-North Myrtle Beach | SC-NC | Metropolitan Statistical Area |
| 10100 | Aberdeen | SD | Micropolitan Statistical Area |
| 39660 | Rapid City | SD | Metropolitan Statistical Area |
| 43620 | Sioux Falls | SD | Metropolitan Statistical Area |
| 47980 | Watertown | SD | Micropolitan Statistical Area |
| 28940 | Knoxville | TN | Metropolitan Statistical Area |
| 34980 | Nashville-Davidson--Murfreesboro--Franklin | TN | Metropolitan Statistical Area |
| 16860 | Chattanooga | TN-GA | Metropolitan Statistical Area |
| 32820 | Memphis | TN-MS-AR | Metropolitan Statistical Area |
| 28700 | Kingsport-Bristol-Bristol | TN-VA | Metropolitan Statistical Area |
| 12420 | Austin-Round Rock | TX | Metropolitan Statistical Area |
| 13140 | Beaumont-Port Arthur | TX | Metropolitan Statistical Area |
| 17780 | College Station-Bryan | TX | Metropolitan Statistical Area |
| 18580 | Corpus Christi | TX | Metropolitan Statistical Area |
| 21340 | El Paso | TX | Metropolitan Statistical Area |
| 19100 | Fort Worth-Arlington | TX | Metropolitan Division |
| 26420 | Houston-The Woodlands-Sugar Land | TX | Metropolitan Statistical Area |
| 41700 | San Antonio-New Braunfels | TX | Metropolitan Statistical Area |
| 48660 | Wichita Falls | TX | Metropolitan Statistical Area |
| 25720 | Heber | UT | Micropolitan Statistical Area |
| 36260 | Ogden-Clearfield | UT | Metropolitan Statistical Area |
| 39340 | Provo-Orem | UT | Metropolitan Statistical Area |
| 41620 | Salt Lake City | UT | Metropolitan Statistical Area |
| 30860 | Logan | UT-ID | Metropolitan Statistical Area |
| 40060 | Richmond | VA | Metropolitan Statistical Area |
| 47260 | Virginia Beach-Norfolk-Newport News | VA-NC | Metropolitan Statistical Area |
| 15540 | Burlington-South Burlington | VT | Metropolitan Statistical Area |
| 42660 | Seattle-Bellevue-Everett | WA | Metropolitan Division |
| 44060 | Spokane-Spokane Valley | WA | Metropolitan Statistical Area |
| 31540 | Madison | WI | Metropolitan Statistical Area |
| 33340 | Milwaukee-Waukesha-West Allis | WI | Metropolitan Statistical Area |
| 16620 | Charleston | WV | Metropolitan Statistical Area |
| 26580 | Huntington-Ashland | WV-KY-OH | Metropolitan Statistical Area |
